# A Semi-Automatic Annotation Approach for Human Activity Recognition

**DOI:** 10.3390/s19030501

**Published:** 2019-01-25

**Authors:** Patrícia Bota, Joana Silva, Duarte Folgado, Hugo Gamboa

**Affiliations:** 1 Associação Fraunhofer Portugal Research, Rua Alfredo Allen 455/461, 4200-135 Porto, Portugal; joana.silva@fraunhofer.pt (J.S.); duarte.folgado@fraunhofer.pt (D.F.); hugo.gamboa@fraunhofer.pt (H.G.); 2 Laboratório de Instrumentação, Engenharia Biomédica e Física da Radiação (LIBPhys-UNL), Departamento de Física, Faculdade de Ciências e Tecnologia, FCT, Universidade Nova de Lisboa, 2829-516 Caparica, Portugal

**Keywords:** human activity recognition, machine learning, active learning, semi-supervised learning, time series, self-training

## Abstract

Modern smartphones and wearables often contain multiple embedded sensors which generate significant amounts of data. This information can be used for body monitoring-based areas such as healthcare, indoor location, user-adaptive recommendations and transportation. The development of Human Activity Recognition (HAR) algorithms involves the collection of a large amount of labelled data which should be annotated by an expert. However, the data annotation process on large datasets is expensive, time consuming and difficult to obtain. The development of a HAR approach which requires low annotation effort and still maintains adequate performance is a relevant challenge. We introduce a Semi-Supervised Active Learning (SSAL) based on Self-Training (ST) approach for Human Activity Recognition to partially automate the annotation process, reducing the annotation effort and the required volume of annotated data to obtain a high performance classifier. Our approach uses a criterion to select the most relevant samples for annotation by the expert and propagate their label to the most confident samples. We present a comprehensive study comparing supervised and unsupervised methods with our approach on two datasets composed of daily living activities. The results showed that it is possible to reduce the required annotated data by more than 89% while still maintaining an accurate model performance.

## 1. Introduction

Over the last years, the technological advances on ubiquitous sensing mechanisms allowed the proliferation of available data, which often is unlabelled. Modern machine learning approaches require large amounts of labelled data to achieve adequate performance. This duality raises a relevant question: How can we simultaneously optimise the process of data annotation and still learn an accurate machine learning model?

Particularly, the Human Activity Recognition (HAR) field has been a source of a large quantity of available data, mostly due to its myriad of applications on real-life scenarios such as healthcare, indoor location, user-adaptive recommendations and transportation [1,2]. According to World Health Organization [3] insufficient physical activity has been identified as the fourth leading risk factor for global mortality, being one of the main causes of several health diseases and correlated with overweight and obesity. HAR research has been trying to mitigate this challenge by monitoring human movement and issuing personalised recommendations in several populations, including the elderly and patients with chronic diseases. On the other hand, the practice of physical exercise is correlated with an increase of cardio-respiratory and muscular fitness, functional health, cognitive functions and improvement of bones and joint health. Additionally, the monitoring of the human movement can be used as a preventive and diagnosis tool for triggering and warning unusual activity such as falls, movement degeneration or cardiac abnormalities. Therefore, HAR has been the subject for numerous research studies over these contexts [2,4,5].

**Figure 1:**
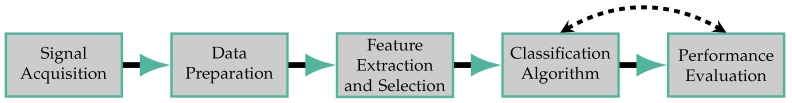
Schematic representation of an Human Activity Recognition (HAR) system architecture.

Most of the latest research work has focused on using machine learning pipelines to accomplish HAR. On a typical HAR framework, as seen in Figure 1 [6], two inputs are often necessary: the raw data and its respective annotation metadata in the form of labels. These labels are often provided by an expert either annotating the data during the acquisition stage or during the course of posterior data evaluation. Human motion information is mapped into raw signals using embed wearable sensors which are often located on different anatomical positions. The data preparation stage consists of reducing the noise arising from the acquisition stage and enhancing the signal characteristics using a set of pre-processing methods. The signal is divided into windows from which features are extracted. Those features, along with the provided labels, are used as the input for the machine learning classifier. Lastly, the classifier predictions are evaluated with the objective of delivering a model capable of issuing correct decisions regarding the daily activities the subject is performing.

To build a representative machine learning model, it is often required the collection of a broad amount of labelled data, which constitutes the ground truth for Supervised Learning (SL) methods. This process aims to train a model capable of correctly generalising into new unlabelled data. The data becomes labelled during a process denoted as annotation, where each sample is mapped to its class. Most of the times, the annotation of data labels must be performed manually by the researcher, becoming a time-consuming, error-prone and expensive task [7]. For instance, let us take as an example the Cityscape dataset [8], which contains stereo video sequences with a total of 5000 high-quality annotated frames. Considering that annotating a single image can take around 1.5 h, to annotate the entire dataset in order to use it as input to a machine learning classifier, it would be required approximately 7500 h. Thus, becoming a fastidious, lengthy task whose quality will higy influence the classification output and whose time could be spent on building the classifier. Therefore, the annotation process might limit real-life application on very large datasets or complex models, where a high volume of data will increase the algorithm’s performance. Under those circumstances, there is a need for the development of a method able to partly automate the data annotation process and reduce considerably its expensive cost.

**Figure 2:**
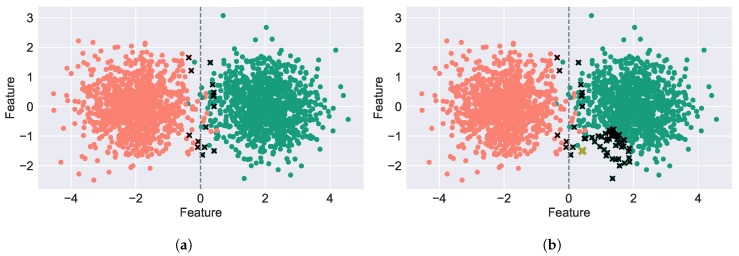
A dataset of 3000 samples illustrating the working principles of Active Learning (AL) and Semi-Supervised Active Learning (SSAL). The samples are illustrated with colours identifying their respective class. The samples selected by the AL for expert annotation are depicted by the ×’s. The grey vertical line denotes the decision boundary between the two classes. (**a**) Active Learning. (**b**) Semi-Supervised Active Learning.

In datasets with significant size, not all samples are equally informative to the classification process and an arbitrary unlabelled example may even be redundant. Active Learning (AL) provides methods to automatically identify the most relevant samples, which are posteriorly queued for expert annotation, that we denote as Oracle, without compromising the model performance. Figure 2a illustrates the behaviour of AL where samples near the decision boundary are selected for annotation since the AL system considers these as the most informative. Additionally, Figure 2b displays the Semi-Supervised Active Learning (SSAL) automatic annotation behaviour where after the most informative sample selected by AL (in yellow) is annotated by the oracle, its nearest samples are automatically annotated (as shown by the × in black).

In this work, we apply a SSAL algorithm for HAR, where we establish criteria to select the most relevant samples for annotation and propagate their label to similar samples and compare it to AL. We present two major contributions: (1) application of SSAL for HAR testing several automatic annotation methods. In literature, studies can be found applying individually both AL and Semi-Supervised Learning (SSL) to HAR. The present work extends these works, combining SSL and AL in the SSAL method and applying it in the context of HAR; (2) in order to accomplish this task with an optimal SSAL system, its detailed steps were evaluated in a comprehensive study of state-of-the-art AL (Query Strategies) QSs, (Stopping Criteria) SCs, and distance functions in the label propagation step. The SSAL method based on Self-Training (ST) applied to HAR data, starting with near zero annotated data, achieved high results on the algorithm’s performance while reducing considerably the annotation effort and automatically annotating a substantial amount of the data.

The remaining of this paper is organised as follows: In Section 2 we present a brief literature review on HAR and AL. Section 3 describes the overall pipeline of the proposed methods. In Section 4, we evaluate our methodology against two datasets comprising real-world HAR data. Lastly, in Section 5, the main achievements of this work are presented along with future work directions.

## 2. Related Work

The discrimination of human activities is often covered either by external or wearable sensors. The former include intelligent homes, where sensors are placed in critical devices and cameras. However, these raise numerous issues, such as privacy, pervasiveness and computational complexity concerns [2,5]. This motivates the use of wearable sensors such as smartphones, since their small size, low cost and non-obtrusiveness allow to integrate them easily into the users’ daily living activities, offering better management of privacy and pervasiveness by giving more control to the user.

On this account, many on-body sensor-based machine learning classification techniques have surged applied in the context of HAR with increasing improved results [2,4,5,9], namely, SL [9,10,11,12,13], Unsupervised Learning (UL) [9,14,15] and more recently, deep learning techniques [16,17,18]. SL is the most common approach for HAR, usually providing the most accurate results. However, SL techniques require high amounts of labelled data, limiting its application on very large dataset scenarios. Over the years a limited number of solutions have been proposed to reduce the amount of the necessary labelled data, namely SSL techniques [7,19,20,21], data automatic annotation [22] or annotation apps [23,24,25]. In the present work, in order to obtain accurate labelled data, an AL system identifies the most important samples to be labelled, therefore, decreasing the amount of necessary labelled data. An AL system is composed of two main parts: the Query System, that selects the most relevant samples from a large unlabelled dataset according to a pre-defined criteria, and the Oracle, an expert annotator to label the selected samples.

In the literature, several techniques for the query system have been proposed. Shahmohammadi et al. [26] applied a Query by Committee and an uncertainty stream-based sampling strategy to a smartwatch-based approach dedicated to HAR. The AL methodology was able to achieve an accuracy of 92%, with a reduction of 46% in the amount of annotated data in comparison to SL. This study allowed to verify that through AL it is possible to create an improved classifier with a reduced number of labelled samples.

To improve the AL accuracy, one of its core points is the applied QS, which establishes a criteria to select the most relevant samples for annotation.

Alembdar et al. [27] presented three methods to measure the classifier’s prediction confidence (i.e., uncertainty) in a sample’s label namely: Least Confident, Margin and Entropy-based Sampling. The proposed QSs outperformed Random Sampling in the reduction of the amount of labelled data, with values from 80% to 66% data reduction.

However, experimental results show that, in some cases, uncertainty-based QSs may tend to select outliers rather than boundary samples [28]. This undesirable behaviour leads to the introduction of bias to the classifier. To overcome this issue, the authors of [28,29,30], used a sampling strategy combining the samples’ uncertainty and the local data density, resulting in the selection of a informative sample inserted in a region of high local density. Since outliers are usually located in low density regions, this procedure minimises the selection of outliers to the data annotation.

In [30] the authors developed a SSAL framework in the context of multivariate time series, using k-Nearest Neighbour (NN) and a k-reverse Nearest Neighbour (rNN) technique to automatically label close neighbours of the newly annotated sample. In the end, for the same amount of initially labelled data, rNN method outperformed the NN method, obtaining higher accuracy, F1-score and percentage of automatically annotated samples.

Lastly, Maja Stikic et al. [31] explored SSL techniques in the context of HAR: Co-Training (two classifiers work on independent data and the most confident predictions of each classifier is used to teach the other), Self-Training (the classifier iteratively increases its training set with its most confidently predicted samples [32]) and AL. Using accelerometer data, Co-Training and ST attained very competitive results, being surpassed by AL using two QSs uncertainty-based functions. Both techniques allowed to significantly reduce the amount of necessary training labelled data and AL was able to outperform the SL technique when trained on the same amount of randomly sampled annotated data.

The literature review allowed verifying the promising results of AL and SSL for HAR in reducing considerably the data annotation effort. The present work extends the state-of-the-art of HAR, combining SSL and AL in a SSAL method applied in the context of HAR. To create an optimal SSAL system, several automatic annotation methods were tested using different distance functions and a comprehensive study regarding state-of-the-art AL QSs and SCs was performed.

## 3. Methods

### 3.1. General Active Learning Strategy

In AL, a QS function selects from a large unlabelled dataset (also referred as pool set) the samples which are more informative to be labelled by the Oracle and added to the classifier’s labelled training set. Algorithm 1 [33] describes the methodology of an AL process. Following the learner’s initialization on the initial training set (*L*), a QS selects the most informative sample ($x^{*}$) from the unlabelled data (*U*) for the oracle to annotate. This process is then repeated iteratively until a stopping criterion is met. Initially *L*<<*U*, however in every iteration, the newly annotated sample $x^{*}$ is removed from *U* and added to *L*, incrementing the labelled train set and consequently, reducing *U*. Hence, in every iteration the learner’s training set expands with informative data and its performance improves [27,34]. The samples considered more informative are usually the samples with the highest gain for the classification process, so that, with a lower amount of labelled data and, therefore, lower data volume and manual annotation effort from the user, it is possible to reach a classification performance similar to a full labelled dataset.

**Algorithm 1** General Active Learning**Input:** initial train set *L*, unlabelled validation set *U*, independent test set *T***Output:** predicted labels for the test set
1:θ←clf.fit(L)▹ Learns model on initial training set2:**while** SC not met **do**3:    selection by QS of the most informative sample: $x^{*}$4:    ask Oracle for $x^{*}$ label5:    $L\leftarrow L\cup x^{*}\;$▹ Increments the model’s training set with $x^{*}$6:    $U\leftarrow U\setminus x^{*}\;$▹ Removes $x^{*}$ from unlabelled samples *U*7:    Θ←clf.fit(L)▹ Updates model8:    return clf.predict(T)▹ Returns predicted labels for the test set9:
**end while**



To obtain a good accuracy in an AL system, there are three main considerations that will be addressed in the forthcoming sub-sections: the initial train set, the QS and the SC.

#### 3.1.1. Initial Train Set

To develop a framework requiring the minimum annotation effort from the user, the initial train set was created with only one sample per class, which was randomly selected and posteriorly removed from the validation set.

#### 3.1.2. Sample Selection Strategy

The second core element of an AL system is the QS, which must be able to select from the unlabelled dataset the sample considered as the most informative. We considered as an informative sample the one that will cause an improvement of the classification performance. Thus, through AL it is possible to optimise the trade-off between the classifier’s performance and the number of labelled samples in its training set. The ability of the AL process to create a representative labelled training set, reaching a higy accurate classification with less labelled data is denoted as Selective Sampling. In contrast, in Passive Learning (PL), samples are chosen randomly from the entire dataset, resulting in a classifier requiring extra annotation effort that does not properly generalise due to its poor and non-representative training data.

Considering a probabilistic model, the classifier prediction output is a U×n matrix, where *U* represents the total number of the unlabelled validation set samples ($X_{U}$= {$x_{1}$, $x_{2}$, …, $x_{U}$}, $x_{i}$ ∈ $R^{m},$ *i* = {1,…,*U*}), and *n* the total number of classes existent in the validation set. Each row is a 1×n vector with the sample’s predicted class probabilities with each cell value given by the prediction posterior probability - $P_{\theta }\left(y_{k}|x_{i}\right)$, k ∈ {0, …, n} under the model θ.

A common metric to evaluate the sample’s usefulness for the classification is to access the classifier’s prediction confidence in that sample’s label [21,27,31], which is given by the classifier’s uncertainty in the sample’s label prediction. In the present work three metrics were studied to evaluate the classifier’s uncertainty, corresponding to three different uncertainty-based selective sampling functions: Least Confident Sampling, Margin Sampling and Entropy Sampling.

- **Least Confident Sampling** [33,34]: Selects the sample whose label the classifier is least certain about, according to the following equation [33,34,35].
  (1) $$\begin{array}{c} x_{LC}^{*}=\underset{x}{arg max}\left(1-P_{\theta }\left(\hat{y}|x\right)\right)\\ \hat{y}=\underset{y}{arg max}\left(P_{\theta }\left(y|x\right)\right) \end{array}$$
  where $\hat{y}$ is the class label which the predictor considers most probable for the sample *x*.
- **Margin Sampling** [33,34]: Selects the sample with the minimum difference (margin) between the prediction probabilities of the first and second most likely classes, according the following equation.
  (2) $$x_{M}^{*}=\underset{x}{arg min}\left(P_{\theta }\left({\hat{y}}_{1}|x\right)-P_{\theta }\left({\hat{y}}_{2}|x\right)\right))$$
  where $\hat{y_{1}}$ and $\hat{y_{2}}$ represent the first and second class labels which the classifier considers as most probable for the sample *x*. Thus, the Margin Sampling QS allows to incorporate into the uncertainty calculation, the probability distribution of one more class label in comparison to Least Confident sampling.
- **Entropy Sampling** [33,34]: Selects the sample with the greatest entropy value, according to the following equation.
  (3) $$x_{E}^{*}=\underset{x}{arg max}\left(-\sum_{k}^{n}P_{\theta }\left(y_{k}|x_{i}\right)\log P_{\theta }\left(y_{k}|x_{i}\right)\right)$$
  where ${\hat{y}}_{i}$ represents the prediction probability of the sample $x_{i}$ belonging to the class $y_{k}$. This method has the advantage of considering the prediction probability for all the class labels, in contrast to the previously mentioned QSs [33,34,35].
  Additionally, in order to create a homogeneous initial training set, a weight $\left(1-p_{l}\right)$ was introduced to the previously mentioned QSs while the training set was less than 1% of the validation set, according to the following equation [36].
  (4) $$x^{*}=\left(1-p_{l}\right)*f$$
  where *f* = {Least Confident Sampling, Entropy Sampling, Margin Sampling} and $p_{l}$ constitutes the percentage of each label in the training set.
  According to the literature [28], a sample with high uncertainty will most likely be an outlier. Thus, to overcome this issue, we tested the Local Density Sampling and the Uncertainty and Local Density Sampling QSs.
- **Local Density Sampling**: Selects the sample with higher representation on the feature space, i.e., located in a high-density region, which is measured by the amount of NNs surrounding the sample, according to the following equation.
  (5) $$x_{LD}^{*}=\underset{x}{arg max}\left(\sum_{i}^{U}\left(\sum_{j}^{k}\frac{1}{1+dist(NN\left(x_{i},x_{j}\right))}\right)\right)$$
  where $x_{i}$ and $x_{j}$ are two samples belonging to the unlabelled samples’ dataset and dist the distance between each sample and its k-NNs. The *k* parameter was empirically set to 5.
- **Uncertainty and Local Density Sampling** [28,30,36]: Obtained through the linear combination between the previously mentioned QSs according to the following equation.
  (6) $$x_{UD}^{*}=\underset{x}{arg max}\left(\alpha f_{0}+\left(1-\alpha \right)f_{1}\right)$$
  where $f_{0}$ is a density weight = {Local Density Sampling}, and $f_{1}$ = {Least Confident Sampling, Margin Sampling, Entropy Sampling}. Setting α to 1, would equal the Uncertainty and Local Density QS to the Local Density QS, while α = 0 to the uncertainty-based QS. The α parameter was set to 0.5 so the QS would choose the most informative sample taking into consideration equally both its local density and its prediction uncertainty.

#### 3.1.3. Stopping Criterion

**Figure 3:**
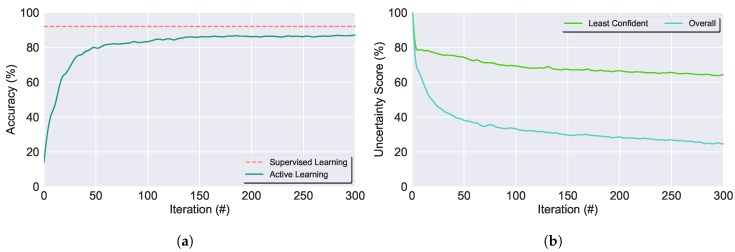
In (**a**) it is shown the AL performance of the initial 300 iterations. The horizontal red line denotes the accuracy average score of Supervised Learning (SL). In (**b**) it is shown the classifier Least Confidence score and Classifier Overall Uncertainty score throughout 300 iterations. (**a**) Least Confidence Uncertainty Score. (**b**) Overall Uncertainty Score.

The last core point to be defined on an AL process is its SC. As it can be seen in Figure 3a, there is an instant during the AL cycle in which the classification’s performance stabilises and, therefore, annotating additional samples will not improve the model’s performance. Hence, the AL process should be ended at this instant, optimising the trade-off between the classifier’s performance and the oracle annotation effort. Therefore, we should guarantee that the AL system must not stop too early, at the cost of resulting in a limited labelled set and under-performing classification, as well that the system does not stop too late either, at the cost of exceeding annotation work. Ideally, we would like to stop when the accuracy of the learner stabilises around its maximum value [37]. However, in a real-life application, we expect to work with unlabelled data, so its ground truth is not available and the accuracy of the classification cannot be obtained.

On this account, with the goal of obtaining a SC applicable to all the methods, QSs and datasets, the following SCs were evaluated:

- **Max-Confidence SC (Max-Conf)** [38]: As previously described, in the Least Confident Sampling it is selected for the oracle to annotate the sample with the highest uncertainty, i.e., the sample that the classifier is least confident in its classification. Moreover, if the selected sample has a low uncertainty score, it is possible to presume that the classifier is able to confidently classify that sample, as well as the remaining samples. Hence, the AL process can be stopped.
- **Overall Uncertainty SC (Over-Unc)** [38]: Similar to Max-Confidence SC, but instead of stopping the AL system if the least confident score is low, it is used the average of the least confident score computed on the remaining unlabelled samples. That is, if this value, denominated overall uncertainty score, shows insignificant low values, we can assume that the classifier has sufficient confidence in the classification of the remaining unlabelled samples and, therefore, the AL cycle can stop.
  Figure 3 shows that the stabilisation of the AL performance overlaps the stabilisation of both the least confident score and the overall uncertainty score. Hence, it was developed a condition to automatically detect whether the scores stabilised based on the mean and standard deviation over a given number of consecutive iterations *S*. The AL process stops when both the two following conditions are verified: $|\mu_{k}-\mu_{k-s}|$≤Δ$\mu_{SC}$ and $|\sigma_{k}-\sigma_{k-s}|$≤Δ$\sigma_{SC}$; k∈{0,2S,…,N}, S=5 and *N* = number of iterations. The Δ$\mu_{SC}$ threshold was obtained through a calibration based on the stabilisation of the classifier accuracy score using the ground truth data.
- **Classification-Change SC (CC)** [37,38]: As discussed in Section 2, uncertainty-based QSs aim to select the most informative samples for the classification, which should correspond to the ones located near decision boundaries. Thus, dictating the class to which each sample is allocated to, therefore, significantly changing the classifier’s performance and its prediction output. Hence, in the CC SC the AL is stopped once decision boundaries samples have been annotated and added to the classifier’s training set. Under these assumptions, alterations in the classifier’s prediction of the unlabelled data labels can be used to infer if the decision boundaries have been changed. Thus, if in two consecutive iterations the classifier’s labels prediction has been constant, then, we can assume that the newly annotated samples are not near a decision boundary but rather inside it, hence, the AL process can be put to an end.
- **Combination Strategy SC**: Consists in a multi-createria-based strategy that combines the prior SCs, namely Overall Uncertainty SC and Classification-Change SC (Over-CC) SC and Max-Confidence Uncertainty SC and Classification-Change SC (Max-CC) SC. The AL is stopped only if both SCs are verified. This method is justified in the cases where the uncertainty score quickly drops to insignificant low values, however, there are inconsistencies in the classifier’s prediction. Thus, the annotation of new samples may result in changes on the decision boundaries and, therefore, on an improvement of the classifier’s performance.

### 3.2. Semi-Supervised Active Learning Framework

As stated in Section 1, there is a need for an annotation technique able to partly automate the annotation process and reduce considerably the annotation cost of constructing a representative labelled dataset in the context of HAR. Thus, with the goal of significantly increasing the amount of available labelled data, we tested the SSAL framework, whose algorithm pipeline is presented in Algorithm 2 [30,39]. The SSAL model is similar to the standard AL framework, however, this method also provides the ability to automatically propagate the annotated label without requiring further inputs from the Oracle.
**Algorithm 2** Semi-Supervised Active Learning**Input:** initial train set *L*, unlabelled validation set *U*, independent test set *T***Output:** predicted labels for the test set1:Θ←clf.fit(L)▹ Learns model on initial training set2:**while** SC not met **do**3:    selection by *Q*, of most informative sample: $x^{*}$4:    ask Oracle for $x^{*}$’s label5:    $L\leftarrow L\cup \{x^{*}\}\;$▹ Augments the model’s training set with $x^{*}$6:    $U\leftarrow U\setminus \{x^{*}\}\;$▹ Removes $x^{*}$ from unlabelled samples7:    Θ←clf.fit(L)▹ Updates model8:    automatically label confident samples *C* in *U*9:    L←L∪{C}▹ Augments the model’s training set with *C*10:    U←U∖{C}▹ Removes *C* from unlabelled samples11:    Θ←clf.fit(L)▹ Updates model12:    return clf.predict(T)▹ Returns predicted labels for the test set13:**end while**

**Figure 4:**
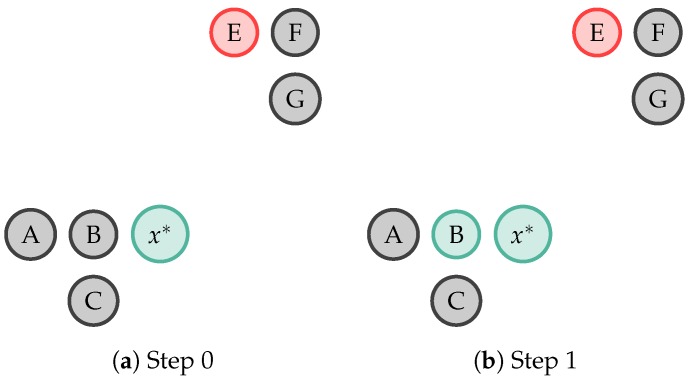
Example of 1-Nearest Neighbour (NN) label propagation, in which the sample $x^{*}$ propagates its label (in this example represented by the colour green) to its 1-NN, the sample *B*. Each circle represents a sample whose colours, green and red, represent two different classes. The samples in grey denote unlabelled samples.

**Figure 5:**
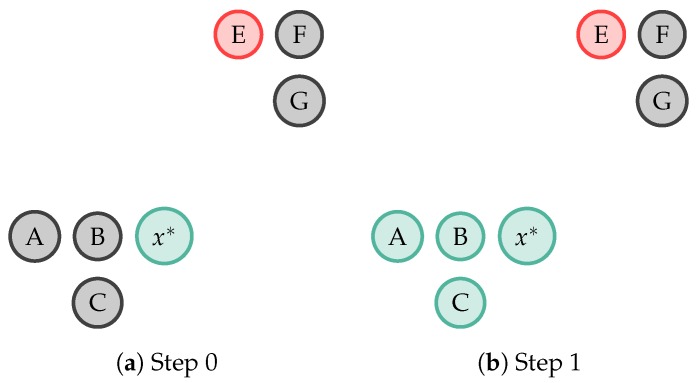
Example of 1-reverse Nearest Neighbour (rNN) label propagation, in which the sample $x^{*}$ propagates its label (in this example represented by the colour green) to the samples to which, regarding the labelled samples, it is their NN, the samples *A*, *B* and *C*. Each circle represents a sample whose colours (green and red) represent two different classes. Samples in grey denote unlabelled samples.

Three SSAL techniques are used:

- **Self-Training Semi-Supervised Active Learning (ST-SSAL)** [31,32,40]: A classifier is trained on the available labelled data and posteriorly tested on the unlabelled data. Validation set samples having the highest prediction confidence score are added to the classifier’s training set and removed from the unlabelled dataset. This process is repeated iteratively as the classifier is re-trained on an increasingly larger and larger training set. Therefore, under the assumption that higy confident predicted labels are correct, the learner uses its own predictions to iteratively teach himself, consequently improving its performance. Hence, a sample will get annotated with $\hat{y}$ if $P_{\theta }\left(\hat{y}|x\right)$ >= $\delta_{ST}$. The $\delta_{ST}$ threshold will influence the amount of propagation and its accuracy. A larger $\delta_{ST}$ will increase the automatic annotation but decrease its accuracy, since the model is less certain in the annotated sample’s label. On the other hand, a smaller $\delta_{ST}$ will decrease the amount of annotation but increase its accuracy, since the few annotations are performed with high certainty. In the present work, $\delta_{ST}$ was empirically set to 0.98 in order to obtain a significant automatic annotation while maintaining a good certainty in the annotation.
- **k-Nearest Neighbour Semi-Supervised Active Learning (k-NN-SSAL)** [30]: The sample selected by the QS ($x^{*}$) propagates its label to its k-NNs. The definition of k (the number of NNs to propagate $x^{*}$’s label) requires a trade-off between the amount of automatic annotation and the addition of error to the system. With a small k, few samples are automatically annotated, but, the ones annotated are done so with a good confidence, as they are close in the feature space. On the other hand, with a higher k, more samples are annotated, however, at the cost of possibly adding error to the classifier, as $x^{*}$ is giving its label to samples at a further distance and therefore, may be wrongly annotated. In the present work, k empirically was set to 5, in order to obtain a significant amount of automatic annotation without compromising the classification accuracy and execution time. Figure 4 depicts the 1-NN label propagation step. Each circle represents a sample whose colours (green and red) represent two different classes. Samples in grey denote unlabelled samples. In this example, the sample $x^{*}$ propagates its label to its 1-NN the sample *B*.
- **k-rNN Semi-Supervised Active Learning (k-rNN-SSAL)** [30]: The sample selected by the QS ($x^{*}$) propagates its label to all the samples to which, regarding the labelled samples, it is their NN and it is within a empirically set distance. For the rNN method, as in [30], k was set to 1 to enhance the label propagation performance. Figure 5 illustrates 1-rNN label propagation step. Thus, in this example, the sample $x^{*}$ propagates its label to the samples *A*, *B* and *C*.

#### Distance Measures

When performing the label propagation step in the NN-SSAL and rNN-SSAL methods, there is a need for a measurement function able to obtain the distance between the different instances. Henceforth, in this section, it is provided four distance measurements. The first two applied in measuring the distance between the feature vector samples and the latter two between time series.

- **Euclidean Distance**: Measures the length of the straight line distance between two samples ($x_{1}$ and $x_{2}$, with dimension *m*) according to the following equation.
  (7) $$\sqrt{\sum_{i=1}^{m}{\left(x_{1}-x_{2}\right)}^{2}}$$
- **Cosine Similarity Distance**: Measures the cosine of the angle between two samples ($x_{1}$ and $x_{2}$) according to the following equation. Cosine similarity ranges between -1 and 1, for opposite and coincident samples, respectively, with the distance value becoming larger as the samples become less similar.
  (8) $$1-\frac{x_{1}\cdot x_{2}}{\left|\right|x_{1}\left|\right|\cdot \left|\right|x_{2}\left|\right|}$$
- **Dynamic Time Warping (DTW)**: Measures the similarity between two time-dependent sequences through a non-linear alignment minimising the distance between both. Moreover, the minimal distance is obtained through the computation of a local cost measure C(S1,S2), where S1:= {$s1_{1}$, $s1_{2}$, …,$s1_{N}$}, S2:= {$s2_{1}$, $s2_{2}$, …,$s2_{M}$} are two time series of length *N* and M;N,*M*∈N, respectively, producing a N×M cost matrix. Where each element corresponds to the Euclidean distance, between each pair of elements in the both sequences. Thus, C(S1,S2), will hold a small value (low cost) if S1 and S2 are similar, or a larger value (high cost) otherwise. Hence, the DTW finds the warping path (*W*) yielding the minimum total cost amount all possible warping paths, by going through the low cost values in the local cost matrix [41,42].
- **Time Alignment Metric (TAM)** [41]: Uses the optimal time alignment obtained by the DTW to infer the intervals when two time series are in phase, advance or in delay in relation to each other. TAM returns a distance metric benefiting series in phase, and penalising when signals are in advance or delay with each other. Thus, resulting in an output value decreasing as the similarity between the two signals increases and increasing otherwise between 0 and 3, the former for signals constantly in phase and the latter for completely out of phase signals. Considering again, two time sequences S1:= {$s1_{1}$, $s1_{2}$, …, $s1_{N}$} and S2:= {$s2_{1}$, $s2_{2}$, …, $s2_{M}$} of length *N* and *M*; *N*, *M*∈N. Assuming S2 is delayed in relation to S1, by a total time $\overset{\leftarrow }{\theta_{S1S2}}$, advanced a total time $\vec{\theta_{S1S2}}$ and in phase by a time $\overset{↔}{\theta_{S1S2}}$. The TAM (Γ) is given by:
  (9) $$\begin{array}{c} \Gamma =\psi_{advance}+\psi_{delay}+\left(1-\psi_{phase}\right),\;\Gamma \in \left\{R_{0}^{+}|\Gamma \in \left[0:3\right]\right\}\\ \psi_{advance}=\frac{\vec{\theta_{S1S2}}}{N},\;\psi_{delay}=\frac{\overset{\leftarrow }{\theta_{S1S2}}}{M},\;\psi_{phase}=\frac{\overset{↔}{\theta_{S1S2}}}{min(N,M)} \end{array}$$

## 4. Results

In this section, we start by introducing the datasets used in this research, followed by an analysis of the performances of the aforementioned methods over several evaluation criteria. All the presented methods were implemented in Python using the modAL framework [43].

### 4.1. Datasets

The performances of the proposed frameworks were evaluated using two real-world datasets fully annotated and class balanced: the public **Human Activity Recognition Using Smartphones Dataset** from University of California Irvine (UCI) [13] and the **Continuous Activities of Daily Living** (CADL) acquired by the authors, whose information is summarised in Table 1. The CADL dataset was obtained continuously, in contrast to UCI dataset, where the activities were segmented. Thus, the CADL was used to provide a validation in a scenario more closely with the real-world requirements. To validate the proposed frameworks, a 10-fold Cross-Validation (CV) was implemented, each fold dividing the dataset into a train and test set. From each train set, one sample per class was chosen randomly to integrate the classifier’s initial training set, while the rest composed the validation set, used to improve the learner in the AL process during 250 iterations. From the 10 folds, the last 5 iterations accuracy score values were averaged to compute the model’s performance accuracy value and its standard deviation.

### 4.2. Signal Processing

**Figure 6:**
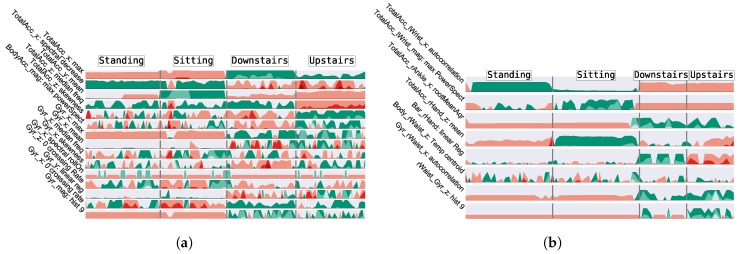
Horizon Plot showing the features and their behaviour along some of the dataset activities. In the y axis, it is presented the information about the sensor, its signal axis and the feature name. The green and red colours denote the signal’s positive and negative values, respectively, with its intensity increasing with the feature’s normalised absolute value and decreasing otherwise. (**a**) UCI dataset. (**b**) CADL dataset.

The data from the CADL dataset was submitted to a band-pass filter with cutoff frequencies of 0.3 Hz and 15 Hz, from which temporal, statistical and frequency domain features were extracted [11,14] from every 5 s window. The data from the UCI dataset was previously pre-processed according to [13]. A forward feature selection method was applied resulting in a feature vector composed of the features presented in Figure 6.

### 4.3. Model Selection

An analysis with common SL and UL techniques was performed with the purpose of finding the optimal technique to incorporate as the learner into the AL process. The respective performance results are shown in Table 2, for the SL and UL methods in accuracy and Adjusted Rand Index (ARI) score, respectively. As observed, Random Forest achieved the highest accuracy in both datasets, 91.4 (2.4)% and 89.1 (4.0)%, for the UCI and CADL dataset, respectively. For the UL methods, Spectral Clustering attained the highest ARI values for both datasets with a score of 57.8 (3.5)% and 61.9 (8.9)%, respectively. It was compared the performance results, for the public UCI dataset, to state-of-the-art researches [44], namely, refs. [10,12,13] who have achieved accuracies of 86%, 96% and 96%, respectively. When training and evaluating the SL method on the same train and test set, it obtained an average accuracy of 89.1 (0.6)%.

### 4.4. QS Analysis

**Figure 7:**
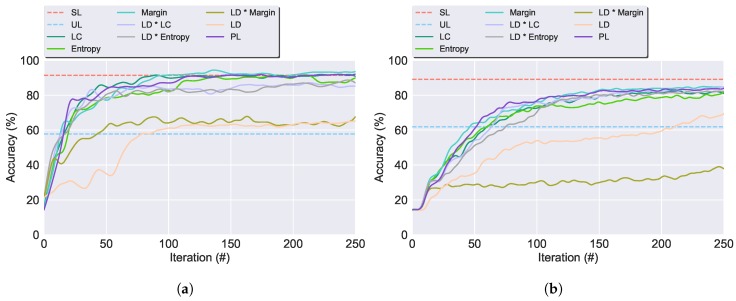
Average increase of the AL classifier’s accuracy for the Query Strategies (QSs) throughout the cycle of iterations. The horizontal lines denote the average accuracy for SL (in red) and UL (in blue). LD denotes the Local Density Sampling and LC the Least Confident Sampling. (**a**) UCI dataset. (**b**) CADL dataset.

**Figure 8:**
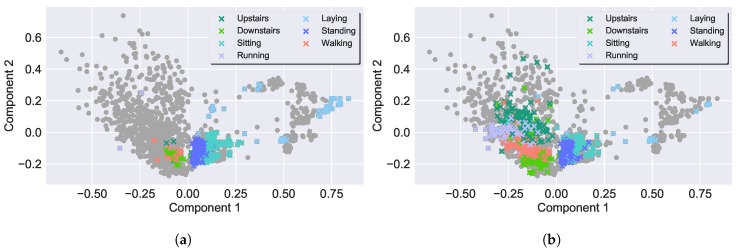
Principal Component Analysis (PCA) of the CADL dataset samples after performing AL for 50 iterations. The classifier’s training set samples are depicted by the ×’s, whose colour identifies their respective class. The darker grey dots represent the unselected samples existent in the validation set. (**a**) Local Density * Margin Sampling. (**b**) Local Density * Least Confident Sampling.

In the current sub-section, the QSs are analysed using the AL framework. This process aims to find the optimal QS, able to obtain the most representative labelled set and consequently attain the highest performance so it could be incorporated into the SSAL frameworks. In Table 3, the QSs are presented against PL (in which the QS randomly selects a sample from the unlabelled dataset), SL and UL. The comparison between the different QSs techniques is performed based on the following criteria:

1. **Accuracy**: The obtained accuracy values from the QSs are very similar and tend to the value obtained by the SL algorithm. This is supported by Figure 7, where it is presented the classifier’s accuracy for the QSs throughout the AL iterations. As expected, overall, the learner becomes more reliable as its training set size increases, resulting in the continuous increase of its accuracy value throughout the iterations. Margin Sampling and Local Density * Least Confident Sampling attain the highest classification’s performances, outperforming PL. However, the difference between AL and PL is low due to two reasons: (1) an initial biased prediction probability due to the classifier very small initial training set; (2) both UCI and CADL datasets are equally balanced. In this circumstance a random selection of samples is enough to create a representative dataset with a few samples from each class. Local Density and Local Density * Margin Sampling, attain the lowest score, not achieving a reasonable performance. These QSs’ low performances are explained by the biased training set, non-representative of the entire dataset distribution under which the classifier operates. As observed in Figure 8a, the density weight causes the preferential selection of activities located in high-density regions, for the deterioration of the remaining as they become unknown for the classifier. Under these circumstances, the classifier does not have a homogeneous training set with sufficient amount of samples from all the class labels from which it can learn to be able to correctly predict all the samples’ labels. Still, with the exception of the aforementioned QSs, the remaining results are in accordance with the literature review [29,30] with the introduction of a density weight to the uncertainty sampling functions avoiding the selection of outliers as observed in Figure 8.
2. **QS Execution Time**: With the exception of the density weighted QSs, in general, the selective sampling functions hold a low execution time. For the density weighted QSs it is observed a significant increase in the QS execution time due to the calculation of the density weight which requires the calculation of each sample’s NNs. This process ultimately increases the algorithm’s computational complexity and execution time. The execution times were obtained using a E3-1285 v6 @ 4.10GHz CPU and 16 GB of RAM.
  Due to the coherent high accuracy performance, surpassing PL, and its low execution time and computational complexity, Margin Sampling was selected as the most suitable QS to be included in both the AL and SSAL frameworks. Hence, forthcoming result presentations on this section were achieved using Margin Sampling. Besides the algorithm’s performance analysis, it is also worth to mention a comparison between the amount of labelled data for SL and AL. From 100% of the validation set annotated in SL, to, approximately 2.8 (0.1)% and 13.9 (0.5)%, for the UCI and CADL dataset, corresponding to the annotation of 250 samples and a reduction of 97.2 (0.1)% and 86.1 (0.5)% in the validation set annotation cost, respectively. These results confirm the applicability of AL in the context of HAR and its efficiency in reducing the annotation effort required to construct a higy confident classifier.

### 4.5. Active Learning Semi-Supervised Analysis

This sub-section aims to present a comparison and select the optimal automatic annotation method. In Table 4 it is shown the methods presented in Section 3.2 compared against techniques previously applied in the context of HAR, described in the literature review, such as AL, PL, SL and UL, replicated in order to verify the model competitiveness.

**Figure 9:**
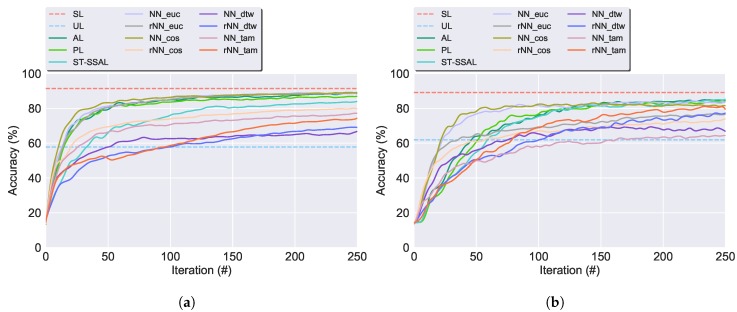
Classifier’s accuracy for the SSAL methods throughout the AL iterations. The horizontal lines denote the 10-CV average accuracy for SL (in red), and UL ARI score (in blue). Following the underscore in the NN and rNN methods: Euc, Cos, Dynamic Time Warping (DTW) and Time Alignment Metric (TAM), denote the distances used. (**a**) UCI dataset. (**b**) CADL dataset.

**Figure 10:**
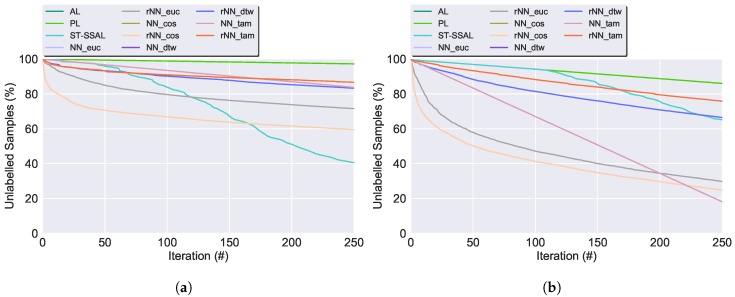
Evolution on the percentage of the validation set unlabelled samples for the SSAL methods throughout the AL cycle iterations. Following the underscore in the NN and rNN methods: Euc, Cos, DTW and TAM, denote the similarity distances used in the respective method. (**a**) UCI dataset. (**b**) CADL dataset.

**Figure 11:**
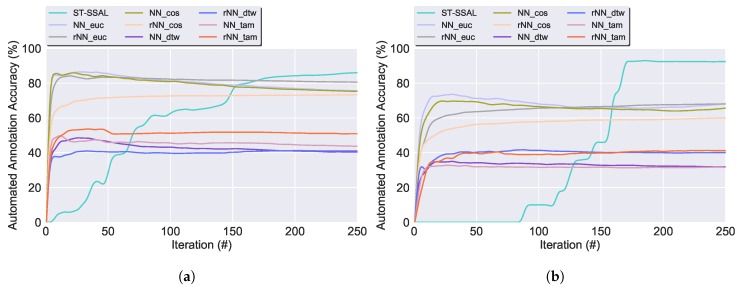
Percentage of correctly automatically annotated samples throughout the AL iterations, for the SSAL methods, using the UCI and the CADL datasets. Following the underscore in the NN and rNN methods: Euc, Cos, DTW and TAM, denote the similarity distances used in the respective method. (**a**) UCI dataset. (**b**) CADL dataset.

1. **Accuracy**: Experimental results demonstrated that with the exception of the SSAL methods using the DTW or TAM distance, the accuracy of the proposed methods converges to the results of the SL technique. Figure 9 presents the classifier’s accuracy for the SSAL methods throughout the AL iterations. For each method, in every iteration the model training set grows, resulting in the increase of the classification’s accuracy.
2. **Automated Annotation Percentage (Aut Ann)**: Consists of the percentage of samples automatically annotated in relation to the total validation set size. Figure 10 displays the evolution on the percentage of the validation set unlabelled samples for the SSAL methods throughout the AL cycle iterations. In the AL and PL, the oracle annotates one sample per iteration, therefore, in Figure 10, both present an overlapping linear decline in the number of unlabelled samples. The NN-SSAL methods annotate six samples per iteration, one by the oracle and five by the automatic annotator, therefore, these show in Figure 10 an overlapping linear decline with higher slope than AL and PL. On the other hand, rNN-SSAL presents a curved decline in the number of unlabelled samples, outperforming the remaining during the first iterations. ST-SSAL displays during the initial iterations an automatic annotation percentage similar to AL and PL, with only the expert annotator labelling new samples and no automatic annotation, since the 0.98 prediction confidence threshold required for automatic annotation is not reached due to the classifier small labelled training set. Once the labelled set becomes representative of the dataset, the 0.98 threshold is reached and ST-SSAL automatic annotation increases exponentially until the unlabelled dataset becomes exhausted, easily surpassing the 5 constantly automatically annotated by the NN-SSAL. On the whole, ST-SSAL attains the highest performance for the UCI dataset, and NN-SSAL for the CADL dataset, the latter closely followed by rNN-SSAL.
3. **Automated Annotation Accuracy (Ann Acc)**: Consists of the percentage of correctly automatically annotated samples. Moreover, Figure 11 presents the evolution throughout the AL process of the automated annotation accuracy for the SSAL methods. As observed, ST-SSAL outperforms the remaining, attaining high results, especially for the latter iterations. ST-SSAL high annotation accuracy on the latter iterations results from the $\delta_{ST}$ threshold required for the automatic annotation to be performed. As noted, this threshold is only reached during the latter iterations when the model training set becomes representative of the dataset and predictions can be performed with high certainty. This fact contrasts with the remaining methods, where higher results are obtained during the first iterations. For the NN-SSAL methods, this is justified by the queried sample propagating its label to closer samples during the first iterations. Whereas in the latter iterations, its closest neighbours start to be already annotated so the sample’s label is given to further away samples. The same is applied to rNN-SSAL, with the stabilisation of the propagation accuracy being accompanied by the stabilisation of the amount of automatic propagation (Figure 10). Additionally, this metric allows to discriminate between the performance of the different distance functions. As it can be seen, the Euclidean distance and Cosine similarity obtained similar results. In contrast to DTW and TAM, presenting a poor percentage of correctly annotated samples, explaining their low classification performance.
4. **Execution time**: AL shows the fastest execution time. The algorithm execution time, allows to favour between the different similarity measures, since the DTW and TAM expensive time and computational complexity, render those algorithms non-applicable to a viable solution. Furthermore, comparing the presented four distance metrics, Euclidean distance presents the lowest time expense and, therefore, was chosen as the most suitable distance metric.

As noted, generally speaking, ST-SSAL outperformed the remaining SSAL methods reaching a higher classification accuracy due to its good performance in the automatic annotation with high certainty. The rNN-SSAL method, although annotating a substantial percentage of the dataset, its lower automatic annotation accuracy performance resulted in the decay of its classification accuracy. To conclude, if we compare ST-SSAL and AL, both methods achieve similar classification performance. However, ST-SSAL was able to annotate a higher volume of data with similar annotation effort without compromising the classification accuracy.

### 4.6. Stopping Criterion Analysis

In this sub-section, we analyse the introduction of a SC to the AL process. Previously, the presented results were based on a pre-defined number of queries (250 queries). As depicted in Figure 9, depending on the dataset some models reach their highest accuracy score quicker than other, stabilising around that value for the forthcoming iterations. Thus, as explained in Section 3.1.3, in order to optimise the trade-off between the classifier’s performance and the expensive training set annotation cost, the number of iterations should be minimised according to the respective algorithm and dataset.

Table 5 presents for both datasets the experimental results for the SSAL methods using the proposed SCs methods in terms of accuracy and below, total number of iterations. Moreover, in the columns SP, for both datasets it is shown the accuracy score for each method in stabilisation and the considered optimal number of iterations at the stopping point. These values were selected in order to achieve a stable accuracy performance with the minimal annotation effort and higher coherency between different folds from the 10-CV (i.e., minimal standard deviation).

The most suitable SC is overall coherent between the different datasets and changes according to the SSAL algorithm. As it can be seen in Table 5, with the introduction of a SC the number of iterations, consequently, the required annotation cost was notably reduced. Therefore, optimising the computational demands of the pipeline. For the ST-SSAL (selected in the previous sub-section as the best performing SSAL method) using the Over-CC SC, an accuracy of 84.5 (4.1)%, F1 score of 82.9 (4.5)% and accuracy of 84.7 (7.2)%, F1 score of 86.3 (6.9)% was attained, with the annotation cost of 214.0 (46.5) and 182.0 (53.9) queries, for the UCI and CADL datasets, respectively, consisting of annotating 2.4 (0.5)% and 10.2 (2.8)% of the validation set. Moreover, the automated annotation along with the manually annotated samples enabled to label 55.8 (11.8)% and 19.1 (13.4)% of the validation set with an accuracy on the automated annotation of 90.5 (4.6)% and 56.7 (46.3)%. Thereupon, the ST-SSAL method allowed to reduce the manual annotation cost on 97.6 (0.6)% and 89.8 (2.8)% for both datasets.

**Figure 12:**
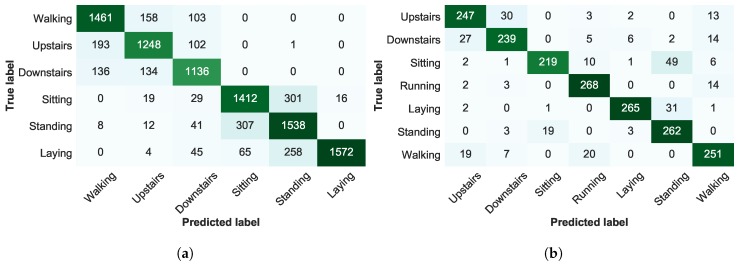
Confusion matrix for the Self-Training Semi-Supervised Active Learning (ST-SSAL) method using the Overall Uncertainty Classification-Change Stopping Criterion (Over-CC SC). (**a**) UCI dataset. (**b**) CADL dataset.

For last, a confusion matrix for the ST-SSAL method using the Over-CC SC is presented in Figure 12, where it is possible to establish conclusions regarding the activities correctly and incorrectly predicted by the classifier. For both datasets, the misclassification was higher between Downstairs/Upstairs and Sitting/Standing. The barometer’s linear regression feature, as it can be seen in Figure 6, presents high distinction between Downstairs/Upstairs, thus, allowed to improve the discrimination between these activities in the CADL dataset. Dynamic activities, due to its distinct motion characteristics and cyclic behaviour presented an overall clear discrimination against static activities.

Lastly, comparing the performance results of the best performing method ST-SSAL method using the Over-CC SC, to state-of-the-art researches for the UCI dataset, namely, [10,12,13] who have achieved accuracies of 86%, 96% and 96%, respectively. When evaluating on the same test set, ST-SSAL obtained an accuracy of 83.2 (4.5)%, after 230.5 (21.9) queries. Therefore, although it did not outperform the aforementioned researches, satisfactory results were achieved, annotating 48.5 (18.1)% of the validation set with an accuracy of 88.0 (5.4)%, and a notable reduction of 96.8 (0.3)% in the training set annotation cost.

## 5. Conclusions

Over the last years, the advances on smartphone and wearable technology allowed the proliferation of their use as unobtrusive and pervasive sensors. The volume of the recorded data by these equipment is significant and poses challenges on the development of traditional machine learning approaches that rely on annotated data to guarantee accurate model performance. The process of annotating a large dataset requires a great effort by the manual annotation of an expert.

Based on the aforementioned challenges in the HAR context, this work addressed a semi-automatic data annotation approach with the goal of optimising the process of data annotation and still be able to learn an accurate machine learning model. Our method relies on two steps: (1) a QS criterion to select the most relevant samples to be labelled by an expert; (2) an automatic method to propagate the annotated sample’s label over similar samples on the entire dataset.

Our main contribution consists of applying SSAL in two HAR datasets, built through a comprehensive study of state-of-the-art QSs and SCs, and the comparison to AL. These methods were evaluated over several automatic annotation strategies based on different distance functions to build an optimal SSAL system with applications for human movement.

Regarding the QS, Margin Sampling achieved the best results in the study performed with AL, reaching an accuracy of 88.4 (2.8)% and 84.8 (0.1)% for UCI and CADL, respectively, and maintaining low computational time.

If we compare ST-SSAL and AL, both methods achieve similar classification performance. However, ST-SSAL was able to annotate a higher volume of data with similar annotation effort, without compromising the classification accuracy. This paper extends the work conducted by [31] on HAR, since it applies ST on the labels previously selected by AL. The ST-SSAL using the Over-CC SC obtained an accuracy of 84.5 (4.1)% and 84.7 (7.2)% for the UCI and CADL datsets, respectively, with a reduction in the Oracle annotation effort on 97.6 (0.6)% and 89.8 (2.8)% of total number of samples for both datasets.

For future work we identified the following research lines: development of a multi-oracle system with non-expert users, allowing the evaluation of the system’s response to any eventual integration of bias in the annotation process; integration of the samples’ estimation annotation cost into the QS, since different samples may present different annotation costs; and the development of an annotation interface which relies on ST-SSAL to facilitate data annotation. The annotation interface can be used to support current methods based on video recordings, with each samples being correlated to a point in time and a recording is present so the user only is required to watch the selected frames given by the QS instead of dedicating extensive hours watching the entire recording.

## Figures and Tables

**Table 1:** University of California Irvine (UCI) dataset and Continuous Activities of Daily Living (CADL) dataset information on: number of users, activities performed, sensors, acquisition device, its position and dataset size.

	Datasets	
	UCI HAR Using Smartphones	Continuous Activities of Daily Living
**N° of Users**	30	12
**Activities**	Laying, sitting, standing, walking, upstairs and downstairs	Laying, sitting, standing, running, walking, upstairs and downstairs
**Sensors**	Accelerometer (50 Hz), gyroscope (50 Hz)	Accelerometer (100 Hz), gyroscope (100 Hz), barometer (30 Hz)
**Device**	Samsung Galaxy S2 (waist)	Samsung S5 (right hand) and wearable sensor (left hand, right ankle and right side of the waist)
**N° of Samples**	10,299	2047

**Table 2:** SL and Unsupervised Learning (UL) methods classification’s performance shown in accuracy and Adjusted Rand Index (ARI) score, respectively. For all listed values it is shown its 10-fold Cross-Validation (CV) average and standard deviation in percentage, the latter between parenthesis. The highest performance is shown in bold for each dataset.

**(a) Supervised Learning**		
	**Dataset**	
**Supervised Learning Method**	**UCI**	**CADL**
**Nearest Neighbours**	91.0 (1.9)	83.6 (3.4)
**Decision Tree**	87.4 (3.5)	83.6 (4.3)
**Random Forest**	**91.4 (2.4)**	**89.1 (4.0)**
**SVM**	90.7 (2.6)	77.6 (3.3)
**AdaBoost**	40.8 (6.8)	54.1 (4.6)
**Naive Bayes**	88.9 (2.9)	75.9 (3.1)
**QDA**	90.8 (2.7)	79.0 (3.7)
**(b) Unsupervised Learning**		
	**Dataset**	
**Unsupervised Learning Method**	**UCI**	**CADL**
**K-Means**	52.1 (4.3)	50.9 (6.1)
**Mini Batch K-Means**	50.7 (5.5)	50.5 (5.3)
**Spectral Clustering**	**57.8 (3.5)**	**61.9 (8.9)**
**Gaussian Mixture**	49.8 (2.7)	58.9 (6.6)
**DBSCAN**	16.4 (7.2)	13.9 (6.5)

**Table 3:** Experimental results for the Query Strategies in terms of: classifier’s accuracy and the QS algorithm’s execution time. For all listed values it is shown its 10-fold CV average and standard deviation, the latter between parenthesis. The best performing algorithm is shown in bold for each dataset.

	UCI Dataset		CADL Dataset	
**Query Strategy**	**Accuracy in %**	**QS Time in s**	**Accuracy in %**	**QS Time in s**
Local Density * Least Confident	87.6 (4.0)	27.2 (5.1)	83.5 (6.7)	0.9 (0.1)
**Least Confident**	87.9 (3.7)	0.1 (0.1)	72.0 (5.0)	0.1 (0.1)
Local Density * Entropy	85.7 (3.7)	22.5 (0.4)	80.3 (7.9)	0.9 (0.1)
**Entropy**	87.1 (2.5)	0.1 (0.1)	70.9 (6.0)	0.1 (0.1)
Local Density * Margin	52.5 (8.6)	22.6 (0.5)	32.8 (8.0)	0.9 (0.1)
**Margin**	**88.4 (2.8)**	**0.1 (0.1)**	**84.8 (7.0)**	**0.1 (0.1)**
**Local Density**	63.6 (5.9)	22.5 (0.4)	68.9 (8.5)	0.9 (0.1)
**Passive Learning**	87.0 (4.5)	0.1 (0.1)	82.0 (7.6)	0.1 (0.1)
**Supervised Learning**	91.4 (2.4)		89.1 (4.0)	
**Unsupervised Learning**	57.8 (3.5)		61.9 (8.9)	

**Table 4:** Experimental results for the SSAL methods: accuracy, automated annotation percentage, automated annotation accuracy and the algorithm’s execution time. For all listed values it is shown its 10-fold CV average and standard deviation, the latter between parenthesis. Following the underscore in the NN and rNN methods: Euc, Cos, DTW and TAM, denote the similarity distances used in the respective method. The best performing algorithm is shown in bold for each dataset.

	UCI Dataset				CADL Dataset			
**Method**	**Accuracy in %**	**Aut Ann in %**	**Ann Acc in %**	**Time in s**	**Accuracy in %**	**Aut Ann in %**	**Ann Acc in %**	**Time in s**
**NN_Euc**	88.1 (2.7)	13.5 (0.1)	76.8 (1.0)	92.3 (9.4)	82.5 (5.7)	68.2 (2.7)	68.2 (1.0)	44.3 (4.8)
**NN_Cos**	89.4 (3.0)	13.5 (0.1)	75.3 (1.4)	860.9 (7.4)	82.8 (8.2)	68.2 (2.7)	64.6 (1.7)	79.1 (4.2)
**NN_DTW**	70.3 (6.0)	13.5 (0.1)	39.7 (1.7)	6772.0 (4.5)	68.4 (5.4)	68.2 (2.7)	30.3 (3.1)	6735.4 (1.3)
**NN_TAM**	75.2 (4.8)	13.5 (0.1)	44.1 (4.0)	6771.4 (5.4)	68.8 (7.3)	68.2 (2.7)	31.6 (1.2)	6735.4 (1.9)
**rNN_Euc**	85.4 (2.7)	33.3 (2.0)	77.3 (3.8)	437.9 (61.9)	74.9 (7.8)	56.6 (1.3)	66.5 (2.7)	60.0 (10.8)
**rNN_Cos**	82.5 (3.6)	37.7 (4.0)	74.7 (5.2)	1165.3 (86.4)	71.3 (8.1)	61.7 (2.5)	59.5 (5.6)	89.7 (11.2)
**rNN_DTW**	65.1 (4.9)	13.7 (1.6)	41.8 (2.9)	6995.4 (74.9)	77.3 (7.1)	20.8 (0.8)	39.9 (1.9)	6739.2 (7.6)
**rNN_TAM**	64.5 (8.4)	11.9 (3.9)	45.7 (4.9)	6968.3 (81.2)	81.8 (8.4)	11.2 (2.0)	41.3 (4.6)	6733.8 (7.3)
**ST-SSAL**	**84.0 (6.3)**	**56.7 (11.6)**	**86.1 (10.5)**	**99.3 (17.5)**	**84.8 (7.0)**	**20.9 (6.9)**	**92.5 (2.7)**	**11.3 (1.0)**
**AL**	88.4 (2.8)			23.6 (5.0)	84.8 (7.0)			12.0 (1.0)
**PL**	87.0 (4.5)			23.2 (1.4)	82.0 (7.6)			10.3 (1.0)
**SL**	91.4 (2.4)			0.7 (0.1)	89.1 (4.0)			0.1 (0.1)
**UL**	57.8 (3.5)			0.3 (0.2)	61.9 (8.9)			0.1 (0.1)

**Table 5:** SC methods accuracy and standard deviation average, acc (std) in percetage. Accuracy score denoted as Acc, and average iterations denoted as N.it over a 10-fold CV on the UCI and CADL datasets. Moreover, for each dataset, under the Stopping Point (SP) columns, the accuracy results in stabilisation are shown and below, the considered optimal number of iterations are shown. The most suitable method is shown in bold for each dataset.

		UCI						CADL					
**Method**		**SP**	**Max-Conf**	**Over-Unc**	**CC**	**Max-CC**	**Over-CC**	**SP**	**Max-Conf**	**Over-Unc**	**CC**	**Max-CC**	**Over-CC**
**NN_Euc**	Acc N.it	85.5 (3.3) 68.5 (26.1)	69.2 (12.5) 20.0 (11.4)	78.7 (8.7) 37.5 (9.5)	73.6 (11.9) 28.9 (12.4)	**82.2 (8.2)** **68.0 (39.3)**	81.5 (9.2) 82.5 (44.1)	81.6 (5.1) 69.6 (38.4)	68.2 (8.9) 44.5 (23.4)	76.7 (6.3) 64.5 (14.3)	57.3 (14.1) 21.2 (8.2)	77.9 (6.0) 123.0 (97.5)	**80.2 (6.4)** **103.0 (73.8)**
**NN_Cos**	Acc N.it	79.6 (7.3) 61.1 (29.7)	70.3 (11.3) 22.0 (13.2)	79.0 (9.6) 41.5 (11.9)	68.8 (13.3) 16.8 (7.6)	81.5 (10.4) 76.5 (47.5)	**82.7 (10.8)** **67.5 (27.0)**	74.6 (15.6) 80.5 (31.4)	62.8 (16.9) 28.0 (10.0)	69.8 (9.9) 52.5 (15.8)	44.2 (20.5) 13.5 (6.8)	74.9 (11.0) 88.5 (65.1)	**78.4 (10.5)** **88.5 (56.9)**
**NN_DTW**	Acc N.it	67.0 (4.3) 250.0 (0.0)	54.3 (6.0) 26.0 (7.4)	47.8 (12.4) 16.0 (2.2)	57.6 (244.7) 244.7 (155.0)	**67.4 (7.4)** **297.7 (105.9)**	58.1 (17.5) 220.4 (158.8)	58.7 (12.5) 117.0 (95.1)	43.9 (7.6) 22.5 (4.5)	33.8 (7.3) 16.0 (5.5)	52.0 (17.0) 215.1 (134.5)	**62.8 (4.0)** **278.3 (79.9)**	64.5 (7.3) 305.3 (11.2)
**NN_TAM**	Acc N.it	77.0 (4.9) 313.2 (78.3)	59.5 (9.0) 37.5 (17.0)	**68.0 (7.9)** **53.5 (10.8)**	63.8 (20.0) 244.6 (153.2)	73.1 (7.0) 318.1 (95.7)	67.3 (12.4) 260.8 (136.7)	62.0 (6.0) 153.0 (86.3)	49.5 (8.0) 47.5 (23.9)	**50.2 (8.4)** **47.5 (14.8)**	60.9 (12.7) 274.9 (90.0)	63.9 (5.2) 305.3 (11.2)	65.8 (5.4) 305.3 (11.2)
**rNN_Euc**	Acc N.it	84.2 (2.3) 92.5 (23.0)	72.6 (12.4) 37.5 (17.6)	**83.7 (3.9)** **97.0 (37.6)**	61.9 (13.4) 15.9 (8.7)	72.6 (13.9) 45.5 (27.4)	84.9 (2.7) 129.4 (52.1)	77.2 (5.9) 198.3 (133.3)	59.9 (14.5) 32.5 (11.8)	**76.9 (9.0)** **174.3 (97.2)**	45.0 (10.8) 14.0 (2.6)	74.9 (7.0) 209.6 (125.4)	78.1 (5.4) 319.1 (92.7)
**rNN_Cos**	Acc N.it	65.5 (9.9) 37.0 (10.4)	**60.6 (11.6)** **31.0 (19.8)**	66.0 (8.2) 37.5 (15.2)	52.7 (10.4) 9.7 (3.5)	65.9 (13.3) 63.5 (71.9)	72.0 (12.1) 66.5 (37.6)	52.7 (16.1) 43.0 (21.5)	53.4 (14.7) 32.0 (11.6)	**60.6 (11.6)** **72.5 (34.2)**	34.2 (17.5) 12.3 (5.6)	53.7 (9.4) 87.4 (101.3)	60.5 (14.4) 165.7 (136.7)
**rNN_DTW**	Acc N.it	38.7 (15.4) 39.0 (27.2)	**44.6 (8.9)** **21.0 (5.9)**	43.0 (6.8) 31.5 (13.2)	29.2 (9.7) 7.4 (0.9)	56.4 (13.9) 156.6 (158.0)	56.1 (14.6) 133.2 (142.5)	41.2 (14.8) 51.0 (32.9)	35.4 (6.9) 25.0 (7.3)	35.9 (6.3) 30.0 (13.0)	23.4 (5.8) 9.3 (4.3)	**56.9 (20.9)** **97.0 (94.2)**	61.2 (18.2) 132.0 (87.2)
**rNN_TAM**	Acc N.it	68.6 (9.4) 290.7 (118.6)	43.1 (10.1) 17.0 (3.7)	48.2 (7.0) 21.0 (5.5)	40.5 (10.2) 12.2 (7.9)	**59.7 (16.7)** **124.3 (137.2)**	50.1 (15.8) 128.2 (146.3)	56.9 (19.6) 77.0 (34.4)	29.8 (7.8) 18.0 (4.6)	27.7 (7.4) 14.5 (5.5)	33.1 (22.6) 22.0 (24.7)	**53.2 (19.0)** **54.0 (29.6)**	47.9 (20.3) 128.2 (146.2)
**ST-SSAL**	Acc N.it	85.2 (3.5) 201.5 (83.8)	48.8 (15.3) 22.5 (9.5)	66.3 (9.9) 61.0 (29.2)	49.1 (18.9) 66.4 (107.1)	75.3 (10.7) 81.0 (41.6)	**84.5 (4.1)** **214.0 (46.5)**	82.8 (6.6) 164.0 (70.1)	33.0 (13.0) 25.0 (10.2)	61.9 (12.1) 67.0 (21.1)	15.0 (0.6) 12.0 (0.0)	76.2 (12.7) 120.0 (28.4)	**84.7 (7.2)** **182.0 (53.9)**
**AL**	Acc N.it	86.1 (2.6) 109.5 (24.4)	60.3 (11.7) 28.5 (10.1)	65.7 (10.5) 38.0 (16.4)	37.4 (11.1) 10.9 (3.9)	**84.0 (4.7)** **98.0 (26.1)**	86.0 (5.6) 97.0 (40.8)	84.6 (7.4) 193.0 (51.0)	51.2 (15.5) 58.0 (17.9)	64.0 (15.0) 118.0 (59.5)	15.0 (0.6) 12.0 (0.0)	**76.8 (9.7)** **116.0 (42.0)**	76.6 (16.5) 139.0 (43.1)
**SL**		91.4 (2.4)						89.1 (4.0)					
**UL**		57.8 (3.5)						61.9 (8.9)					
